# The impact of China’s clean energy market on the market connectivity of rare earth industrial chain

**DOI:** 10.1371/journal.pone.0342223

**Published:** 2026-02-23

**Authors:** Junhui Li, Yanqiong Zhao, Shiquan Dou, Yongguang Zhu, Deyi Xu

**Affiliations:** 1 School of Economics and Management, China University of Geosciences, Wuhan, China; 2 Mineral Resource Strategy and Policy Research Center, China University of Geosciences, Wuhan, China; Universiti Malaysia Sabah, MALAYSIA

## Abstract

The accelerating global transition to clean energy underscores the critical role of rare earth elements in renewable technologies. As the world’s largest producer of clean energy and rare earth minerals, China’s market dynamics are pivotal to the stability and connectivity of the rare earth industrial chain. This study investigates the interplay between China’s clean energy market and the connectivity of its rare earth industry chain using a Time-Varying Parameter Vector Autoregression model, as well as Autoregressive Distributed Lag and Nonlinear Autoregressive Distributed Lag models. The results reveal significant short-term spillover effects from clean energy market shocks, which enhance connectivity within the rare earth industry chain, while long-term impacts weaken these interactions as markets stabilize. Asymmetric effects are pronounced, with the photovoltaic sector exerting a particularly significant influence on rare earth market connectivity. These findings highlight the dynamic and nonlinear linkages between clean energy and rare earth markets, providing valuable insights for policymakers and investors to ensure industrial chain resilience and support sustainable development.

## Introduction

The global climate crisis represents one of the 21st century’s most urgent challenges, compelling governments and organizations to expedite the clean energy transition. The International Energy Agency’s World Energy Outlook 2023 forecasts renewables reaching nearly 60% of global electricity by 2030 [1]. This shift is essential for climate mitigation and green growth [2,3]. As the largest carbon emitter and fossil fuel consumer, China is central to this landscape, committing to peak emissions by 2030 and carbon neutrality by 2060.

These pledges demand widespread adoption of wind, solar, and electric vehicle technologies, where rare earth elements—prized for their unique magnetic and chemical properties—are indispensable [4,5]. Yet, with China dominating over 70% of global production [6], the supply chain’s concentration heightens risks of price volatility and instability, potentially impeding clean energy progress [7]. This policy-driven context directly informs our empirical objectives: to analyze how clean energy market dynamics influence rare earth industry connectivity, guiding strategies for energy security and sustainable transition.

The rapid expansion of China’s clean energy market has surged demand for rare earth materials, influenced by policies, technological advancements, market dynamics, and geopolitical factors [8]. For instance, the photovoltaic (PV) sector’s boom may initially strengthen rare earth market connectivity in the short term, yet long-term industrial adjustments could mitigate these effects. Similarly, the electric vehicle (EV) and wind energy sectors introduce unique dynamics, heightening overall uncertainties in the rare earth industry.

Despite extensive research on the interplay between rare earths and clean energy, existing studies often rely on static models that overlook the time-varying nature of risk transmission and fail to account for asymmetric responses to positive versus negative market shocks [7,9]. This leaves a critical gap in understanding how fluctuating clean energy demands dynamically reshape industry chain connectivity over time. Our study addresses this by employing an advanced dynamic modeling framework, with a targeted sectoral focus on PV, EV, and wind energy, and incorporating recent data periods to reveal evolving interactions that prior work has not fully explored.

To examine these interactions, this study combines Time-Varying Parameter Vector Autoregression (TVP-VAR), Autoregressive Distributed Lag (ARDL), and Nonlinear Autoregressive Distributed Lag (NARDL) models. This multifaceted approach is justified conceptually as follows: TVP-VAR allows for the capture of evolving dynamics and time-varying risk transmissions across the industry chain; ARDL explores long-run equilibrium relationships between clean energy fluctuations and rare earth connectivity; and NARDL specifically uncovers asymmetric effects, distinguishing between responses to positive and negative market shocks. Together, these techniques provide a robust framework for analyzing short- and long-term interactions. By incorporating key variables such as PV, EV, and wind energy indices, and controlling for external factors like geopolitical risks (GPR) and economic policy uncertainty (EPU), this study investigates the mechanisms through which the clean energy market drives connectivity in the rare earth industry [10].

The primary objectives of this research are:

1. To investigate the transmission of risks across the upstream, midstream, and downstream segments of the rare earth industry chain in response to dynamic fluctuations in the clean energy market.
2. To examine whether the clean energy market exerts asymmetric effects on the connectivity of the rare earth industry chain.
3. To propose optimized policies and investment strategies for stabilizing the rare earth industrial chain amid evolving demands from the clean energy sector.

Based on these objectives and the identified research gap, we propose the following hypotheses to guide the analysis and provide clear motivation:

H1: Dynamic fluctuations in China’s clean energy market will positively enhance risk transmission and connectivity across the rare earth industry chain in the short run, as immediate demand surges amplify spillovers, but weaken them in the long run due to market adaptations and stabilizations.

H2: The clean energy market will exhibit asymmetric effects on rare earth connectivity, with negative shocks (e.g., declines in market indices) having a stronger disruptive impact than positive shocks, reflecting supply rigidities and investor risk aversion.

H3: Targeted policies, particularly those supporting the PV sector, will optimize rare earth chain stability by mitigating asymmetries, aligning with China’s goals for sustainable energy transitions.

The empirical findings reveal a high average connectivity of 31.2% in rare earth markets, with dynamic spillovers peaking during policy shifts like the 2015 tariff cancellation and 2023 green initiatives, but fluctuating amid events such as COVID-19 disruptions. ARDL results show short-run positive effects from clean energy indices on connectivity, transitioning to long-run negatives for sola, while NARDL uncovers asymmetries, with PV positive shocks reducing long-run connectivity more than negatives, underscoring PV’s stabilizing yet saturation-prone role in the chain.

This research contributes to the literature in several ways. First, it provides a systematic framework for analyzing the relationship between the clean energy market and the rare earth industrial chain, addressing gaps in the current understanding of dynamic and asymmetric effects [9]. Second, it offers valuable insights for policymakers, such as designing targeted policies to support the PV market and managing strategic reserves to ensure industrial chain stability [10,11]. Third, it equips investors with a quantitative basis for assessing market risks and optimizing their portfolios in the clean energy and rare earth sectors, fostering sustainable industrial development.

## Literature review

### Industrial chain dynamics in the rare earth sector

China’s rare earth industry relies on a comprehensive industrial chain, spanning from upstream resource extraction to downstream technological applications, creating a distinct industrial advantage. However, despite this complete industrial chain, China’s position within the global rare earth value chain has yet to be fully realized. This is particularly evident in intermediate stages such as refining, where China faces increasing competitive pressure, as the U.S. and its allies gradually challenge its dominance in the global market. These countries are actively rebuilding the rare earth industrial chain through policies, legal frameworks, and equity transactions to reduce their dependency on China, particularly in refining, while simultaneously enhancing autonomy in downstream industries, especially in electric vehicles and renewable energy.

Existing literature highlights the asymmetry of China’s competitive advantage within the global rare earth value chain. Shuai et al. (2022) utilized a projection pursuit model to analyze China’s comparative advantage in rare earth products within the industrial chain, emphasizing that China holds a strong competitive edge in midstream and downstream sectors but lacks competitiveness in upstream mining activities [12]. Zuo et al. (2022) analyzed global rare earth trade data, noting a trend towards integration in the upstream and downstream sectors, while the midstream sector shows diversification [13]. However, these studies primarily focus on trade and production efficiency, without exploring in depth the effects of geopolitical factors, international sanctions, or changes in investment patterns on the rare earth industrial chain. Leng et al. (2021) employed social network analysis (SNA) to reveal China’s increasing technological innovation capacity in the rare earth industry, while still exhibiting a “strong upstream, weak downstream” structure [14]. While this finding enhances the understanding of China’s dominance in rare earth mining, it does not address how international competitors, particularly the U.S., use policies to influence this industry structure. Zhang et al. (2022) and Xia et al. (2023) examined competition across various stages of the rare earth industrial chain and found that competition is most intense in downstream markets, particularly in sectors related to clean energy technologies [15,16]. However, existing studies have not sufficiently addressed domestic policy changes in China, especially in response to international pressures, and how these factors influence the adjustment of China’s rare earth industrial chain. These dynamics significantly affect the competitive landscape of the global rare earth market.

Recent studies further emphasize supply chain integration dynamics. For instance, Severson et al. (2023) conducted an integrated supply chain analysis for rare earth elements under global electrification scenarios, highlighting the need for diversified sourcing to mitigate risks in clean energy transitions [17]. Similarly, Xu et al. (2025) examined interdependence in rare earth element supply between China and the United States using a multi-layer network approach, revealing how trade linkages stabilize global chains despite geopolitical tensions [18].

While existing research provides valuable insights into the rare earth industrial chain, there remains a significant gap in understanding how global geopolitical shifts impact the competitiveness of the rare earth industry chain. However, these studies often employ static models such as vector error correction models (VECM) or basic regression analyses, lacking time-varying perspectives that account for evolving geopolitical and market shifts over time [12,13]. Therefore, this study will employ a TVP-VAR model to quantify the dynamic relationships between different stages of the rare earth industrial chain, addressing these methodological gaps by incorporating time-varying parameters to analyze the effects of Chinese policies and international market changes. This theme of industrial chain dynamics underscores the need for models that capture evolving inter-segment relationships, setting the stage for examining linkages with broader markets such as clean energy.

### Clean energy–rare earth linkages and energy-finance interactions

With the global energy transition, the share of renewable energy in global energy supply has steadily increased, with rare earth elements playing a critical role in this transformation. In recent years, the scale of clean energy equipment construction has significantly grown, particularly in the wind and solar sectors. According to the International Energy Agency, global renewable energy capacity additions reached approximately 510 GW in 2023, a nearly 50% increase compared to the previous year [19]. China’s newly added PV capacity in 2023 was comparable to the total global installation in 2022, while wind power capacity grew by 66%, with PV panels accounting for two-thirds of the global renewable energy capacity additions.

Post-pandemic economic recovery and the response to the global energy crisis have greatly accelerated investments in clean energy. From 2021 to 2023, the annual growth rate of clean energy investment reached 24%, far exceeding the 15% growth in fossil fuel sectors [20]. This surge has directly boosted demand for rare earths, which are critical raw materials in clean energy technologies, including wind power, solar energy, and electric vehicles [21].

Building on these linkages, several studies have delved into energy-finance interactions, such as price spillovers and market correlations. Several studies have explored the relationship between clean energy and the rare earth market. Ding et al. (2024) examined the relationship between global warming, renewable energy consumption, and the rare earth market, finding that during the COVID-19 pandemic, global warming and renewable energy utilization had a positive short-term effect on the rare earth market [22]. However, this study did not investigate how policy changes might affect long-term demand dynamics in the rare earth market. Apergis and Apergis (2017) noted a temporal correlation between rare earth prices and renewable energy, but did not explore in depth the GPR impacting the rare earth market [23]. Fritz and Schiefer (2009) analyzed the impact of rare earth metal prices on clean energy indices using a multi-factor market model, revealing a negative correlation between rare earth prices and the performance of certain clean energy stocks during periods of price increase [24]. While this finding is of practical significance, it does not adequately consider the broader impacts of global economic fluctuations on clean energy investment patterns. Madaleno et al. (2023) studied the spillover effects between rare earth elements and renewable energy, noting that these effects vary with market conditions and time frames [25]. This research provides valuable insights into the relationship between rare earth markets and renewable energy market fluctuations, but has not thoroughly explored the influence of geopolitical tensions on these spillover effects. Recent research has advanced understanding of clean energy market spillovers and dynamic connectedness. Ye et al. (2023) analyzed time-varying spillovers between rare earth prices and renewable energy sectors, showing heightened connectedness during market volatility periods [26]. Gao et al. (2024) explored higher-order moments connectedness between rare earth and clean energy markets from a multiscale perspective, uncovering asymmetric risk transmissions that vary across short- and long-term horizons [27]. While insightful, many of these analyses rely on static vector autoregression (VAR) models or correlation-based approaches, which overlook time-varying connectedness and asymmetric effects in spillovers [23,24]. This limitation hinders a full understanding of how GPR and economic fluctuations dynamically influence the rare earth-clean energy nexus. These themes highlight the interconnectedness of energy transitions and financial markets, yet gaps persist in dynamic analyses, which this review addresses next through theoretical frameworks.

### Theoretical framework for industrial chain and market dynamics

Several theoretical channels have been proposed in the literature to influence industrial industrial chains, including the bullwhip, capacity-constraining, cost-compensating, and contagion effects [28–31]. These theories provide a robust foundation for analyzing how exogenous shocks influence the interactions within the rare earth market. A ubiquitous challenge in industrial chain design is the “bullwhip effect” [28], wherein small fluctuations in downstream customer demand result in significant alterations in upstream production levels [32–35]. The capacity constraint effect reflects the fact that changes in the production and market demand for upstream raw materials can significantly affect the maximum production capacity of midstream and downstream products within the industrial chain [30,36]. The cost compensation effect suggests that price changes in midstream and downstream products may influence the market prices of upstream raw materials through subsidies to production costs [31]. The market contagion effect refers to the potential for external shocks to impact the prices of mid- and downstream products within the industrial chain due to the interconnectedness of the market [37–39].

**Fig 1:**
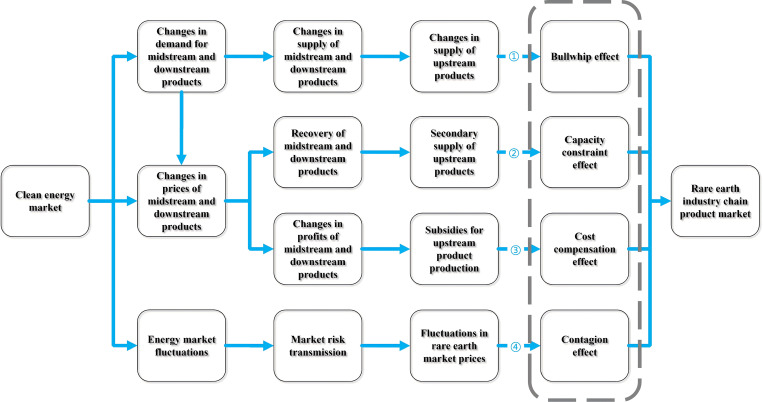
Mechanisms and pathways for clean energy market to influence the product market of rare earths industrial chain.

Building upon the aforementioned models, this paper examines the pathways through which the clean energy market influences the interconnectedness among product markets in the rare earth industry chain (see Fig 1). The rare earth market is exposed to potential risks from the evolution of the clean energy market, which can trigger exogenous shocks to the prices, demand, and supply of products within the rare earth industry chain. These effects represent distinct pathways through which the clean energy market impacts the interconnectedness of rare earth industry chain product markets.

Pathway 1 describes the route through which the clean energy market induces the bullwhip effect in the rare earth industry chain. Growth in the clean energy market serves as a major driver of demand for rare earth minerals [24]. Consequently, to meet the increased demand, there is a need to expand the supply of downstream products in the industrial chain, implying that upstream production must also increase, further amplifying the bullwhip effect (Pathway 1).

However, as demand grows, the prices of downstream products in the rare earth industry chain are likely to rise, allowing downstream manufacturers to realize higher profits. This situation incentivizes rare earth recycling companies to increase the recycling of discarded rare earth products, thus boosting the secondary supply of upstream rare earth products. This initiative reduces the dependency of downstream products on primary upstream raw materials [40,41], thereby alleviating the capacity constraint effect (Pathway 2).

Pathways 3 and 4 illustrate how the clean energy market affects the cost compensation and contagion effects within the price connectivity of the rare earth industry chain. As clean energy technologies become more widespread, the strategic importance and economic value of rare earth metals are further emphasized. For companies that simultaneously produce upstream, midstream, and downstream products in the rare earth industry chain, an increase in downstream product prices can lead to higher production profits, which can then subsidize the relatively low returns from upstream raw material production. This implies that price changes in downstream products, driven by the clean energy market, may affect the market prices and profitability of upstream products (Pathway 3). Moreover, as fluctuations in the energy market affect the rare earth market [25,42], the volatility in the clean energy market can cause price fluctuations in the rare earth industry chain through market contagion effects (Pathway 4).

In summary, while existing literature illuminates industrial chain dynamics, clean energy–rare earth linkages, and energy-finance interactions, significant unknowns persist, including the time-varying impacts of geopolitical shifts, asymmetric spillovers in market connectedness, and the integration of supply chains under electrification pressures. Methodological gaps, such as reliance on static VAR or VECM models, further limit insights into evolving relationships. This study addresses these voids by employing advanced time-varying models like TVP-VAR and NARDL to empirically analyze dynamic risk transmissions, asymmetric effects, and policy influences within the rare earth industry chain, offering a more nuanced understanding of its interplay with the clean energy market.

Theoretically, this research contributes by advancing key concepts such as asymmetry—distinguishing responses to positive (e.g., demand surges) versus negative (e.g., policy restrictions) shocks—and time-varying effects, capturing regime changes like those induced by geopolitical tensions or electrification policies. Empirically, it innovates through a novel integration of TVP-VAR for evolving spillovers, ARDL for long-run equilibria, and NARDL for nonlinear asymmetries, applied to a unique dataset of monthly rare earth returns and clean energy indices from 2015–2023, enabling robust quantification of market dynamics that prior static approaches overlook.

## Methodology and data

### Methodology

#### Time-varying parameter vector autoregression (TVP-VAR).

Our research applied the TVP-VAR methodology to explore the evolving spillovers among product markets at each phase of the Chinese PV industry chain. The TVP-VAR model was originally put forward by Primiceri, n.d. (2005) [43]. This model presents a distinctive benefit, as it can capture nonlinear relationships that vary over time among economic variables. It achieves this by permitting variations in the coefficients and variance covariance matrices over time [44–46]. As per Antonakakis et al. (2020) [47], this model has two key advantages. First, it helps to settle the problem of selecting the best scrolling window size. Second, it avoids to loss valuable observations and hence is also suitable for short samples.

The TVP-VAR model, as proposed by Primiceri, n.d. (2005) [43], can be expressed as:


$$y_{t}=c_{t}+\sum {\mathrm{\backslash nolimits}}_{k=\text{1}}^{p}B_{k,t}y_{t-k}+u_{t}$$ (1)

where $y_{t}$ is an N × 1 vector of observed variables, $c_{t}$ is an N × 1 time-varying intercept vector, $B_{k,t}$ are N × N time-varying coefficient matrices for lags k = 1 to p, and $u_{t}\sim N(\text{0},\Omega_{t})$ is an N × 1 error vector with time-varying covariance matrix $\Omega_{t}$. The covariance matrix is decomposed as $\Omega_{t}=A_{t}^{-\text{1}}H_{t}(A_{t}^{-\text{1}}{)}^{\prime }$ where $A_{t}$ is a lower triangular matrix with ones on the diagonal, and $H_{t}$ is a diagonal matrix of stochastic volatilities.

Following Diebold and Yılmaz (2014) [48], the TVP-VAR is transformed into its vector moving average (VMA) representation based on Wold’s theorem:


$$y_{t}=\sum {\mathrm{\backslash nolimits}}_{h=\text{0}}^{\infty }\Phi_{h,t}u_{t-h}$$ (2)

where $\Phi_{h,t}$ are N × N time-varying coefficient matrices in the VMA representation.

The generalized impulse response function (GIRF), denoted as $\psi_{ij,t}^{g}(H)$, measures the response of variable j to a shock in variable i over horizon H:


$$\psi_{ij,t}^{g}(H)=E(y_{t+H}\;|\;u_{i,t}=\delta_{i,t},I_{t-\text{1}})-E(y_{t+H}\;|\;I_{t-\text{1}})$$ (3)

where $\delta_{i,t}=(\sigma_{jj,t}{)}^{-\text{1}/\text{2}}$, and $\sigma_{jj,t}$ is the j-th diagonal element of $\Omega_{t}$. The GIRF is scaled as:


$$\theta_{ij,t}^{g}(H)=\sigma_{jj,t}^{-\text{1}/\text{2}}\Phi_{H,t}\Omega_{t}e_{i}$$ (4)

with $e_{i}$ an N × 1 selection vector with 1 in the i-th position and 0 elsewhere.

The generalized forecast error variance decomposition (GFEVD), $\theta_{ij,t}^{g}(H)$, represents the proportion of the H-step-ahead forecast error variance of variable i attributable to shocks in variable j:


$$\phi_{ij,t}^{g}(H)=\frac{\sigma_{ii,t}^{-\text{1}}\sum_{h=\text{0}}^{H-\text{1}}(e_{i}^{\prime }\Phi_{h,t}\Omega_{t}e_{j}{)}^{\text{2}}}{\sum_{h=\text{0}}^{H-\text{1}}\left(e_{i}^{\prime }\Phi_{h,t}\Omega_{t}\Phi_{h,t}^{\prime }e_{i}\right)}$$ (5)

Normalized as,


$${\tilde{\phi }}_{ij,t}^{g}(H)=\frac{\phi_{ij,t}^{g}(H)}{\sum_{j=\text{1}}^{N}\phi_{ij,t}^{g}(H)}$$ (6)

Subsequently, we compute the aggregate spillover index using GFEVD as follows:


$$S_{t}^{g}(H)=\frac{\text{1}\text{0}\text{0}}{N}\sum {\mathrm{\backslash nolimits}}_{i\neq j}{\tilde{\phi }}_{ij,t}^{g}(H)$$ (7)

The spillover index present in Eq. (7) usually demonstrates the spilling over of shocks from one variable towards other variables under examination. However, to conduct a more comprehensive analysis of directional linkages, this study categorizes them into three types: directional spillovers passed on to others; directional spillovers received from others; and net spillovers (Table 1).

**Table 1:** Spillover indices and their interpretations.

Index	Calculation Formula	Interpretation
Total spillovers	$S_{t}^{g}(H)=\frac{\text{1}\text{0}\text{0}}{N}\sum {\mathrm{\backslash nolimits}}_{i\neq j}{\tilde{\phi }}_{ij,t}^{g}(H)$	Assess the extent to which shocks to one variable propagate to other variables.
Directional spillovers to others	$S_{i\to j,t}^{g}(H)=\frac{\text{1}\text{0}\text{0}}{N}\sum {\mathrm{\backslash nolimits}}_{j\neq i}{\tilde{\phi }}_{ji,t}^{g}(H)$	Measure the impact of the shock that is transmitted from variable i to other variables j.
Directional spillovers from others	$S_{j\to i,t}^{g}(H)=\frac{\text{1}\text{0}\text{0}}{N}\sum {\mathrm{\backslash nolimits}}_{j\neq i}{\tilde{\phi }}_{ij,t}^{g}(H)$	Measurement variable i is susceptible to disturbances from other variables j.
Net spillovers	$S_{i,t}^{g}(H)=S_{i\to j,t}^{g}(H)-S_{j\to i,t}^{g}(H)$	Measures the effect of variable i’s influence on the entire network of variables.

First, we analyze the directional spillovers that are passed on to other variables (j) when variable (i) sends its shocks as demonstrated in Eq. (8):


$$S_{i\to j,t}^{g}(H)=\frac{\text{1}\text{0}\text{0}}{N}\sum {\mathrm{\backslash nolimits}}_{j\neq i}{\tilde{\phi }}_{ji,t}^{g}(H)$$ (8)

Second, we examine the impacts of shocks that variable i experiences from other variables j in Eq. (9).


$$S_{j\to i,t}^{g}(H)=\frac{\text{1}\text{0}\text{0}}{N}\sum {\mathrm{\backslash nolimits}}_{j\neq i}{\tilde{\phi }}_{ij,t}^{g}(H)$$ (9)

Finally, subtract Eq. (9) from Eq. (8) to obtain a net spillover. To calculate the net spillover, we deduct the directional spillover received from other parties from the directional spillover transmitted to them.


$$S_{i,t}^{g}(H)=S_{i\to j,t}^{g}(H)-S_{j\to i,t}^{g}(H)$$ (10)

Eq. (10) illustrates how variable i affects the network being analyzed. Hence, a positive $S_{i,t}^{g}(H)$ indicates that variable i exerts a stronger impact on the network than the network has on itself. Conversely, a negative $S_{i,t}^{g}(H)$ indicates that the network motivates variable i.

We selected the TVP-VAR model because it is well-suited for capturing the dynamic and evolving nature of interactions among the product markets of the rare earth industry chain, especially given the rapid changes in the clean energy market and the external shocks that might impact this chain. The model allows us to track how the relationships between variables shift over time, which is crucial when dealing with time series data that is subject to changing economic conditions [47].

Furthermore, the TVP-VAR model is advantageous because it addresses the limitations inherent in models that use fixed parameters, providing a more flexible framework that can adapt to time-varying relationships. The ability to estimate time-varying parameters enables us to better understand the evolution of spillovers across different phases of the industry chain, especially under changing external conditions, such as shifts in policy or market dynamics.

However, limitations of the TVP-VAR model must be acknowledged. One key limitation is the complexity of estimation, as time-varying parameters require more computational resources and are prone to overfitting when the sample size is small. This can lead to potential biases if not properly managed. Additionally, while the model is highly flexible, it may struggle to adequately model nonlinearities or capture long-term equilibrium relationships that are often of interest in industrial chain analyses. In this context, we complement the TVP-VAR with other models, such as ARDL and NARDL, to investigate both long-term and short-term effects.

#### Autoregressive distributed lag model (ARDL) and nonlinear autoregressive distributed lag model (NARDL).

To further investigate the asymmetric effects of China’s clean energy market on the connectivity of the rare earth industry chain, this study employs both the ARDL and NARDL models. The ARDL model is particularly advantageous due to its flexibility in handling small sample sizes and accommodating variables integrated at different levels, such as I(0) or I(1). This model allows for the simultaneous estimation of long-run and short-run parameters, providing valuable insights into the dynamic equilibrium of the system [49].

However, while the ARDL model is widely used, it assumes symmetric relationships between variables. Such an assumption may not be appropriate in contexts where variables exhibit different impacts depending on whether they increase or decrease. Financial time series, including those related to the rare earth and clean energy sectors, often demonstrate nonlinear behaviors, where a positive shock may yield a different effect compared to a negative shock. To better capture these potential nonlinearities, we introduce the NARDL model, developed by Shin et al. (2014) [50]. The NARDL model facilitates the estimation of asymmetric short- and long-run effects, offering a nuanced understanding of how positive and negative changes in the clean energy market differentially influence the rare earth industry chain.

Empirically, NARDL is justified for asymmetric economic relationships, as it decomposes variables into positive and negative partial sums to capture threshold effects often seen in commodity markets [51]. For instance, in energy economics, NARDL has revealed asymmetric pass-throughs in oil prices to inflation or growth, where negative shocks (price falls) have stronger impacts due to sticky adjustments [50]. In this study, BDS test results confirming nonlinearity in series like NEI further support NARDL, enabling robust analysis of how clean energy declines disrupt rare earth connectivity more than increases enhance it, addressing gaps in linear models.

The ARDL model is specified as [49]:


$$\Delta y_{t}=\alpha +\rho y_{t-\text{1}}+\theta^{\prime }x_{t-\text{1}}+\sum {\mathrm{\backslash nolimits}}_{i=\text{1}}^{p-\text{1}}\lambda_{i}\Delta y_{t-i}+\sum {\mathrm{\backslash nolimits}}_{i=\text{0}}^{q-\text{1}}\pi_{i}^{\prime }\Delta x_{t-i}+\varepsilon_{t}$$ (11)

where $y_{t}$ is the dependent variable, $x_{t}$ is a k × 1 vector of regressors, ρ is the error correction term, θ are long-run coefficients, $\lambda_{i}$ and $\pi_{i}$ are short-run coefficients, and $\varepsilon_{t}$ is the error term.

To illustrate, we constructed an ARDL model using the New Energy Index (NEI) to analyze its impact on the total connectivity (TC) of China’s rare earth industry chain:


$$\begin{array}{cc} \Delta TC_{t}=\; & \alpha +\rho TC_{t-\text{1}}+\theta_{\text{1}}NEI_{t-\text{1}}+\theta_{\text{2}}GPR_{t-\text{1}}+\theta_{\text{3}}EPU_{t-\text{1}}+\theta_{\text{4}}TPU_{t-\text{1}}+\sum {\mathrm{\backslash nolimits}}_{i=\text{1}}^{p-\text{1}}\lambda_{i}\Delta TC_{t-i}\\ & \;\;\;+\sum {\mathrm{\backslash nolimits}}_{i=\text{0}}^{q-\text{1}}\left(\pi_{\text{1}i}\Delta NEI_{t-i}+\pi_{\text{2}i}\Delta GPR_{t-i}+\pi_{\text{3}i}\Delta EPU_{t-i}+\pi_{\text{4}i}\Delta TPU_{t-i}\right)+\varepsilon_{t} \end{array}$$ (12)

Here, TC represents the total connectivity index of China’s rare earth industry chain, NEI is the new energy index, GPR is the geopolitical risk index, EPU is the economic policy uncertainty index, and TPU is the trade policy uncertainty index.

In the NARDL framework, asymmetry is introduced by decomposing explanatory variables (e.g., NEI) into positive and negative components using partial sum processes:


$$x_{t}^{+}=\sum {\mathrm{\backslash nolimits}}_{j=\text{1}}^{t}\Delta x_{j}^{+}=\sum {\mathrm{\backslash nolimits}}_{j=\text{1}}^{t}max\left(\Delta x_{j},\text{0}\right)$$ (13)

$$x_{t}^{-}=\sum {\mathrm{\backslash nolimits}}_{j=\text{1}}^{t}\Delta x_{j}^{-}=\sum {\mathrm{\backslash nolimits}}_{j=\text{1}}^{t}min\left(\Delta x_{j},\text{0}\right)$$ (14)

The NARDL model is:


$$\Delta y_{t}=\;\alpha +\rho y_{t-\text{1}}+\theta^{+}x_{t-\text{1}}^{+}+\theta^{-}x_{t-\text{1}}^{-}+\sum {\mathrm{\backslash nolimits}}_{i=\text{1}}^{p-\text{1}}\lambda_{i}\Delta y_{t-i}\;\;\;+\sum {\mathrm{\backslash nolimits}}_{i=\text{0}}^{q-\text{1}}\left(\pi_{i}^{+}\Delta x_{t-i}^{+}+\pi_{i}^{-}\Delta x_{t-i}^{-}\right)+\varepsilon_{t}$$ (15)

This decomposition captures how increases (positive shocks) and decreases (negative shocks) in clean energy indices differentially affect rare earth connectivity. Theoretically, asymmetry is expected because positive shocks (e.g., policy incentives boosting EV/PV demand) may enhance chain integration linearly, while negative shocks (e.g., trade restrictions) could amplify disruptions due to supply chain rigidities and investor risk aversion, consistent with nonlinear behaviors in commodity markets [50].

For example, using the NEI, we constructed a NARDL model to explore how positive and negative changes in the NEI influence the rare earth industry chain’s connectivity:


$$\begin{array}{cc} \Delta TC_{t}=\; & \alpha +\rho TC_{t-\text{1}}+\theta^{+}NEI_{t-\text{1}}^{+}+\theta^{-}NEI_{t-\text{1}}^{-}+\theta_{\text{2}}GPR_{t-\text{1}}+\theta_{\text{3}}EPU_{t-\text{1}}+\theta_{\text{4}}TPU_{t-\text{1}}\\ & \;\;\;\;\;+\sum {\mathrm{\backslash nolimits}}_{i=\text{1}}^{p-\text{1}}\lambda_{i}\Delta TC_{t-i}+\sum {\mathrm{\backslash nolimits}}_{i=\text{0}}^{q-\text{1}}\left(\pi_{i}^{+}\Delta NEI_{t-i}^{+}+\pi_{i}^{-}\Delta NEI_{t-i}^{-}+\pi_{\text{2}i}\Delta GPR_{t-i}+\pi_{\text{3}i}\Delta EPU_{t-i}+\pi_{\text{4}i}\Delta TPU_{t-i}\right)+\varepsilon_{t} \end{array}$$ (16)

The formula is as follows: $\theta^{+}$ represents long-run coefficient of positive changes in NEI, $\theta^{-}$ represents long-run coefficient of negative changes in NEI, $\pi_{i}^{+}$ denotes short-run coefficient of positive changes in NEI, $\pi_{i}^{-}$ denotes short-run coefficient of negative changes in NEI, $\sum_{i=\text{0}}^{q-\text{1}}\pi_{i}^{+}$ is cumulative short-run impact of positive changes, and $\sum_{i=\text{0}}^{q-\text{1}}\pi_{i}^{-}$ is cumulative short-run impact of negative changes.

While the ARDL and NARDL models offer powerful tools for analyzing relationships, they are not without limitations. The ARDL model assumes linearity, which may oversimplify relationships in contexts characterized by large, nonlinear changes. Both models also require assumptions about cointegration and stationarity, which may not always hold in real-world data. The NARDL model, while addressing asymmetry, introduces additional complexity by decomposing variables into positive and negative changes, making interpretation more challenging and increasing the risk of overfitting.

By integrating the ARDL and NARDL models, this study captures both dynamic spillovers and asymmetric effects, offering a comprehensive framework for analyzing the interactions between China’s clean energy market and the rare earth industry chain.

The selected models complement each other as follows: TVP-VAR captures time-varying spillovers and regime changes (e.g., policy shifts), addressing RQ1 and H1 on dynamic risk transmission; ARDL establishes symmetric long-run equilibria, supporting RQ3 and H3 on policy optimization; NARDL uncovers asymmetries in positive/negative shocks, central to RQ2 and H2 on differential effects. This synergy is significant for the hypotheses, as it allows testing evolving (TVP-VAR), balanced (ARDL), and nonlinear (NARDL) relationships in volatile markets [43,49,50].

**Fig 2:**
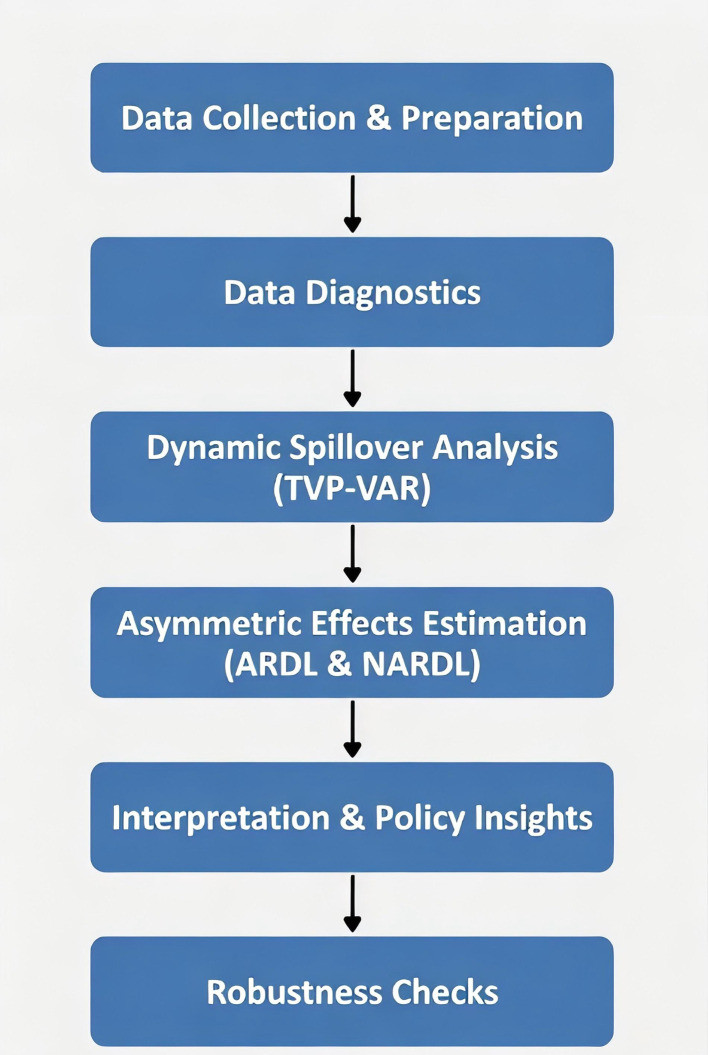
Methodological flowchart.

To visually summarize how these models interrelate in the analytical process, Fig 2 provides a step-by-step methodological flowchart. This diagram illustrates the sequential pipeline, starting from data preparation and diagnostics, proceeding through TVP-VAR for capturing time-varying dynamics and spillovers, followed by ARDL and NARDL for examining symmetric and asymmetric effects, and concluding with interpretation and robustness validation.

### Data

The dataset comprises 17 variables, primarily monthly time series data spanning from February 2015 to August 2023 (Table 2). Rare earth industry chain products include upstream (Praseodymium-Neodymium Oxide (PNO), Dysprosium Oxide (DO), Neodymium Oxide (NO), Praseodymium Oxide (PO)), midstream (Dysprosium Metal (DM), Neodymium Metal (NM), Praseodymium Metal (PM), Pr-Nd Alloy (PNA), Dy-Fe Alloy (DFA)), and downstream (Nd-Fe-B (NFB)) items, sourced from the Shanghai Nonferrous Metals Network via the Wind database. Clean energy market proxies are the CSI New Energy Index (NEI), CSI New Energy Vehicle Index (NEVI), CSI Wind Power Industry Index (WIND), and CSI Photovoltaic Industry Index (SOLAR), obtained from the CSI Index website (www.csindex.com.cn). Control variables include the Geopolitical Risk Index (GPR), Economic Policy Uncertainty Index (EPU), and Trade Policy Uncertainty Index (TPU), accessed from the Economic Policy Uncertainty website (www.policyuncertainty.com). GPR captures interstate tensions and threats, potentially affecting commodity market information flows. EPU and TPU, per Davis et al. (2019), quantify policy-related uncertainties impacting metal market connectivity [52].

**Table 2:** Detailed description of variables.

Variable	Symbol	Data Source	Unit	Frequency	Transformation
Praseodymium-Neodymium Oxide	PNO	Wind Database (Shanghai Nonferrous Metals Network)	CNY/ton	Monthly	Logarithmic returns: $r_{it}=\text{100}*\left(ln\left(p_{t}\right)-ln\left(p_{t-\text{1}}\right)\right)$
Dysprosium Oxide	DO	Wind Database (Shanghai Nonferrous Metals Network)	CNY/ton	Monthly	Logarithmic returns: $r_{it}=\text{100}*\left(ln\left(p_{t}\right)-ln\left(p_{t-\text{1}}\right)\right)$
Neodymium Oxide	NO	Wind Database (Shanghai Nonferrous Metals Network)	CNY/ton	Monthly	Logarithmic returns: $r_{it}=\text{100}*\left(ln\left(p_{t}\right)-ln\left(p_{t-\text{1}}\right)\right)$
Praseodymium Oxide	PO	Wind Database (Shanghai Nonferrous Metals Network)	CNY/ton	Monthly	Logarithmic returns: $r_{it}=\text{100}*\left(ln\left(p_{t}\right)-ln\left(p_{t-\text{1}}\right)\right)$
Dysprosium Metal	DM	Wind Database (Shanghai Nonferrous Metals Network)	CNY/ton	Monthly	Logarithmic returns: $r_{it}=\text{100}*\left(ln\left(p_{t}\right)-ln\left(p_{t-\text{1}}\right)\right)$
Neodymium Metal	NM	Wind Database (Shanghai Nonferrous Metals Network)	CNY/ton	Monthly	Logarithmic returns: $r_{it}=\text{100}*\left(ln\left(p_{t}\right)-ln\left(p_{t-\text{1}}\right)\right)$
Praseodymium Metal	PM	Wind Database (Shanghai Nonferrous Metals Network)	CNY/ton	Monthly	Logarithmic returns: $r_{it}=\text{100}*\left(ln\left(p_{t}\right)-ln\left(p_{t-\text{1}}\right)\right)$
Pr-Nd Alloy	PNA	Wind Database (Shanghai Nonferrous Metals Network)	CNY/ton	Monthly	Logarithmic returns: $r_{it}=\text{100}*\left(ln\left(p_{t}\right)-ln\left(p_{t-\text{1}}\right)\right)$
Dy-Fe Alloy	DFA	Wind Database (Shanghai Nonferrous Metals Network)	CNY/ton	Monthly	Logarithmic returns: $r_{it}=\text{100}*\left(ln\left(p_{t}\right)-ln\left(p_{t-\text{1}}\right)\right)$
Nd-Fe-B	NFB	Wind Database (Shanghai Nonferrous Metals Network)	CNY/ton	Monthly	Logarithmic returns: $r_{it}=\text{100}*\left(ln\left(p_{t}\right)-ln\left(p_{t-\text{1}}\right)\right)$
New Energy Index	NEI	CSI Index Website (www.csindex.com.cn)	Index points	Monthly	Logarithmic returns: $r_{it}=\text{100}*\left(ln\left(p_{t}\right)-ln\left(p_{t-\text{1}}\right)\right)$
New Energy Vehicle Index	NEVI	CSI Index Website (www.csindex.com.cn)	Index points	Monthly	Logarithmic returns: $r_{it}=\text{100}*\left(ln\left(p_{t}\right)-ln\left(p_{t-\text{1}}\right)\right)$
Wind Power Industry Index	WIND	CSI Index Website (www.csindex.com.cn)	Index points	Monthly	Logarithmic returns: $r_{it}=\text{100}*\left(ln\left(p_{t}\right)-ln\left(p_{t-\text{1}}\right)\right)$
Photovoltaic Industry Index	SOLAR	CSI Index Website (www.csindex.com.cn)	Index points	Monthly	Logarithmic returns: $r_{it}=\text{100}*\left(ln\left(p_{t}\right)-ln\left(p_{t-\text{1}}\right)\right)$
Geopolitical Risk Index	GPR	Economic Policy Uncertainty Website (www.policyuncertainty.com)	Index value	Monthly	No transformation
Economic Policy Uncertainty Index	EPU	Economic Policy Uncertainty Website (www.policyuncertainty.com)	Index value	Monthly	No transformation
Trade Policy Uncertainty Index	TPU	Economic Policy Uncertainty Website (www.policyuncertainty.com)	Index value	Monthly	No transformation

The sample covers monthly data from February 2015 to August 2023, selected primarily due to data availability from the specified sources. This period encompasses key policy milestones and structural shifts relevant to the rare earth and clean energy sectors, including China’s 2015 rare earth resource tax reforms, the 2020 Export Control Law implementation (which influenced price surges), and the 2022 Russia-Ukraine conflict’s impact on global commodity markets. These events allow for analysis of dynamic spillovers amid evolving geopolitical and economic uncertainties, aligning with the study’s focus on time-varying interactions.

Prior to estimation, we conducted unit root tests to assess stationarity: Augmented Dickey-Fuller (ADF), Phillips-Perron (PP), and Kwiatkowski-Phillips-Schmidt-Shin (KPSS). Results indicated mixed integration orders, with most variables I(1) at levels but I(0) after first differencing, justifying ARDL/NARDL use. Cointegration was tested via ARDL bounds approach [49], confirming long-run relationships where F-statistics exceeded critical bounds.

To detect nonlinear dependencies, we applied the BDS test [53], which rejects the null of independent and identical distribution (iid) for key series like TC and NEI, confirming nonlinearity and supporting the use of asymmetric models like NARDL.

Model lag orders were selected using Akaike Information Criterion (AIC) for TVP-VAR to optimize fit, and Schwarz Information Criterion (SIC) for ARDL/NARDL to favor parsimony, with maximum lags of 4 based on data length.

**Fig 3:**
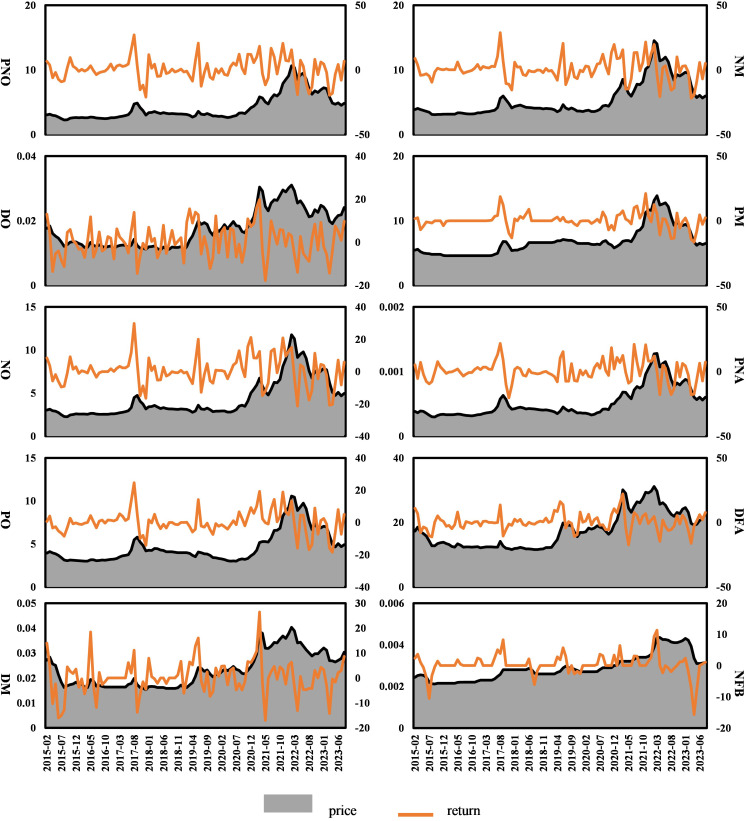
Fluctuations in prices and returns of rare earth industry chain products from February 2015 to August 2023.

Table 3 provides descriptive statistics for each variable. We calculated the returns for each market i at time t using the formula$r_{it}=\text{100}*\left(ln\left(p_{t}\right)-ln\left(p_{t-\text{1}}\right)\right)$, where p represents the spot prices of Chinese rare earth products and new energy market indices. Using this return data, we explored the correlations among the products in the rare earth industrial chain. The monthly prices and return changes for PNO, DO, NO, PO, DM, NM, PM, PNA, DFA, and NFB are illustrated in Fig 3.

**Table 3:** Descriptive statistics for all variables.

Variable	Obs	Mean	Std.Dev.	Min	Max	Ske.	Kur.	JB test^a^
PNO	103	0.514	8.778	−21.129	27.006	0.059	3.721	2.294
DO	103	0.429	7.626	−17.751	19.856	0.094	2.598	0.845
NO	103	0.560	8.998	−21.387	29.855	0.146	3.912	3.936
PO	103	0.220	7.133	−18.493	24.595	0.314	4.776	15.226***
DM	103	0.247	6.938	−17.026	26.424	0.290	4.968	18.062***
NM	103	0.502	8.802	−21.850	28.792	0.223	4.085	5.907*
PM	103	0.182	5.803	−16.571	21.064	0.186	5.791	34.017***
PNA	103	0.487	8.156	−20.360	21.772	0.056	3.617	1.689
DFA	103	0.372	6.493	−17.306	22.122	0.238	4.249	7.669**
NFB	103	0.273	3.296	−15.755	11.348	−0.936	10.011	225.999***
NEI	103	0.560	8.933	−34.108	21.881	−0.586	4.860	20.739***
NEVI	103	0.745	9.351	−31.203	20.113	−0.428	3.581	4.593
WIND	103	0.381	9.465	−32.753	22.851	−0.548	4.752	18.325***
SOLAR	103	0.620	9.363	−33.071	24.294	−0.611	4.716	19.045***
GPR	103	0.807	0.354	0.270	2.560	1.365	7.459	117.342***
EPU	103	263.054	124.114	60.200	661.800	0.620	3.410	7.319**
TPU	103	323.730	269.714	8.000	1425.200	1.563	6.067	82.295***

This table shows descriptive statistics of monthly returns of 10 rare earth product markets, 4 new energy market indices, and 3 control variables from February 2015 to August 2023.

^a^The normality of the series is tested using the Jarque-Bera statistic test. Significance levels of 1%, 5% and 10% denoted by ***, ** and * (the same below).

In December 2020, with the formal implementation of China’s Export Control Law, prices of rare earth-related products began to soar rapidly, reaching their peak in March 2022. From March 2022, the impact of the Russia-Ukraine conflict, which resulted in a spike in commodity prices, partially reduced the profit margins of rare earth companies. This has resulted in reduced demand for rare earths, resulting in a gradual decline in their prices. Fig 3 illustrates significant fluctuations in the returns of all markets, particularly in 2022. Table 3 shows that among all rare earth products, NO exhibits the highest average returns and volatility. Except for NFB, the positive skewness coefficients indicate that nine series are right-skewed. In the new energy markets, NEVI exhibits the highest average returns, whereas SOLAR demonstrates the highest volatility. All variables in the new energy markets exhibit right-skewed distributions. Regarding kurtosis coefficients, except for DO, all variables demonstrate noticeable leptokurtic distributions. Apart from PNO, DO, NO, PNA and NEVI, the Jarque-Bera statistics show elevated values which reject the null hypothesis and indicate that all variables are not normally distributed.

### Robustness checks

To ensure result stability, we conducted sub-sample analyses: (1) pre-2020 (February 2015–November 2020) to isolate pre-Export Control Law effects, and (2) post-2020 (December 2020–August 2023) amid heightened volatility. Additionally, alternative proxies were tested, such as replacing GPR with the International Country Risk Guide (ICRG) political risk index and EPU with global EPU variants. Across specifications, spillover patterns and asymmetric effects remained consistent, with minor variations in magnitude during volatile periods, affirming the models’ reliability.

## Empirical results and discussion

### TVP-VAR results: Static and dynamic connectivity

This study examines the connectivity of rare earth industry chain product markets using the TVP-VAR method, with the optimal lag order determined by the AIC over a forecast range of 10. Initially, conventional spillover methods were applied to evaluate spillover effects and provide a deeper understanding of the interconnections among rare earth industry chain product markets. Table 4 presents the static connectivity indices for various variables.

**Table 4:** Static spillover indices.

	PNO	DO	NO	PO	DM	NM	PM	PNA	DFA	NFB	FROM^a^
PNO		14.666	24.104	23.173	14.448	23.588	18.244	23.265	15.905	13.835	171.229
DO	14.364		13.635	12.807	20.791	13.859	7.398	11.513	23.157	7.424	124.947
NO	24.085	14.215		22.694	13.646	24.442	17.375	22.378	16.044	14.962	169.841
PO	22.644	12.256	22.294		12.406	22.197	20.001	20.907	13.986	13.695	160.385
DM	14.599	20.286	13.970	13.527		13.959	6.209	12.074	22.221	11.246	128.090
NM	23.478	14.057	24.372	22.551	13.708		17.970	21.916	16.101	15.092	169.245
PM	16.859	7.087	15.818	18.614	5.771	16.473		16.175	7.868	12.322	116.987
PNA	23.256	10.738	22.430	22.134	11.711	22.270	18.297		13.028	15.218	159.081
DFA	15.869	22.754	16.085	14.616	22.214	16.188	8.473	13.389		10.086	139.673
NFB	8.315	1.857	8.862	9.282	4.563	9.020	9.589	9.185	3.962		64.634
TO^b^	163.469	117.915	161.570	159.398	119.258	161.995	123.556	150.802	132.270	113.879	TC
NET^c^	−7.760	−7.032	−8.271	−0.987	−8.832	−7.249	6.569	−8.279	−7.403	49.244	31.203

The table displays the outcomes of the overall connectivity within rare earth industry chain.

^a^“FROM” indicates spillovers received from other variables.

^b^“TO” indicates spillovers to other variables.

^c^The net connectivity “NET” is calculated as “TO” minus “FROM”. “TC” represents the total connectivity index.

The total connectivity among markets is 31.2%, indicating substantial interactions and a lack of market independence within the rare earth industry chain. This aligns with Madaleno et al. (2023) [26], who found varying spillovers between rare earths and renewables, but our time-varying focus reveals higher connectivity during policy surges, extending their static analysis by capturing regime shifts. PM and NFB emerge as the primary net contributors to spillover effects. Key findings include:

Upstream Market: PNO generates the largest spillover effect on other products, dominating the upstream market alongside NO and PO. These products are pivotal in transmitting risks across the industrial chain, consistent with the bullwhip effect where upstream demand fluctuations amplify downstream volatility [28].

Midstream Market: NM exhibits a spillover effect of 162%, significantly influencing NO. This highlights the midstream market’s role as a critical node for risk propagation and interaction.

Downstream Market: The downstream market is primarily impacted by PM and receives significant spillovers from upstream products. PNO, NO, and PNA are the major recipients of market spillovers, while DM is the largest net recipient of risk premiums.

Product Connectivity: The strongest connectivity among rare earth products occurs between NO and NM, indicative of efficient information transmission within these markets. In contrast, the weakest connectivity is observed between DO and NFB, reflecting limited interaction between these products.

The static connectivity analysis provides an overview of the average level of connectivity, but it does not capture the dynamic fluctuations in connectivity over time. This section presents the time-varying connectivity of China’s rare earth industry chain product market.

**Fig 4:**
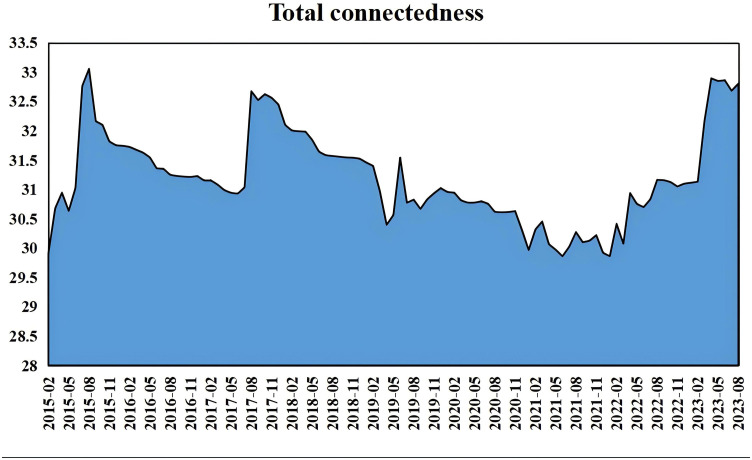
Time-varying total connectivity of China’s rare earth industry chain product market. Note: This figure illustrates the dynamic total spillover index (in percentage terms) among rare earth product returns from February 2015 to August 2023. The y-axis represents the connectivity index (%), ranging from 25% to 35%, while the x-axis denotes time on a monthly scale with labeled years (2015−2023). Blue shaded areas highlight significant structural shift periods, such as policy announcements (e.g., May 2015 tariff cancellation) and external shocks (e.g., 2020 COVID-19), where connectivity exceeds 32% (1 standard deviation above mean). Peaks indicate heightened risk transmission due to clean energy demand surges.

Fig 4 illustrates the total connectivity of returns among rare earth industry chain product markets over time. The total connectivity fluctuates between 30% and 33%, indicating notable spillover effects among these markets. Due to exogenous shocks affecting the Chinese rare earth market, the spillover effects exhibit considerable time-varying characteristics.

Key Moments in Dynamic Connectivity and Structural Shifts:

May 2015 (Policy Shift): The Chinese Ministry of Commerce’s cancellation of rare earth export tariffs, amid a global commodity recovery, spiked connectivity to ~32%, as investors capitalized on improved trade, boosting upstream-midstream linkages (see Fig 3 peak). This policy-driven peak echoes Hanif et al. (2023)’s COVID spillovers [42], but our results show stronger upstream transmission in recovery phases, highlighting rare earths’ sensitivity to domestic policies over global shocks.

July 2017 (Renewable Energy Push): The National Energy Administration’s “13th Five-Year Plan” for renewables drove a surge to 33%, reflecting heightened demand from clean energy growth, with midstream products like NM transmitting risks downstream. Consistent with Bouri et al. (2023)’s volatility connectedness [54], but our study emphasizes midstream roles in renewables push, differing by quantifying 33% peaks tied to plan implementation.

2018-2019 (Trade War Effects): Connectivity dipped to ~30% during US-China trade tensions, indicating structural shifts from tariffs disrupting supply chains, increasing volatility in upstream products like PNO (Fig 4 shows net receivers).

2020−2021 (COVID-19 Disruption): Amid pandemic supply chain breaks, connectivity fluctuated wildly, with downstream NFB as a net transmitter, highlighting economic resilience but policy needs for diversification.

April 2023 (Green Recovery): Post-COVID “Two Sessions” and green development white paper pushed connectivity to 32.89%, signaling renewed investor confidence in rare earths for energy transition.

**Fig 5:**
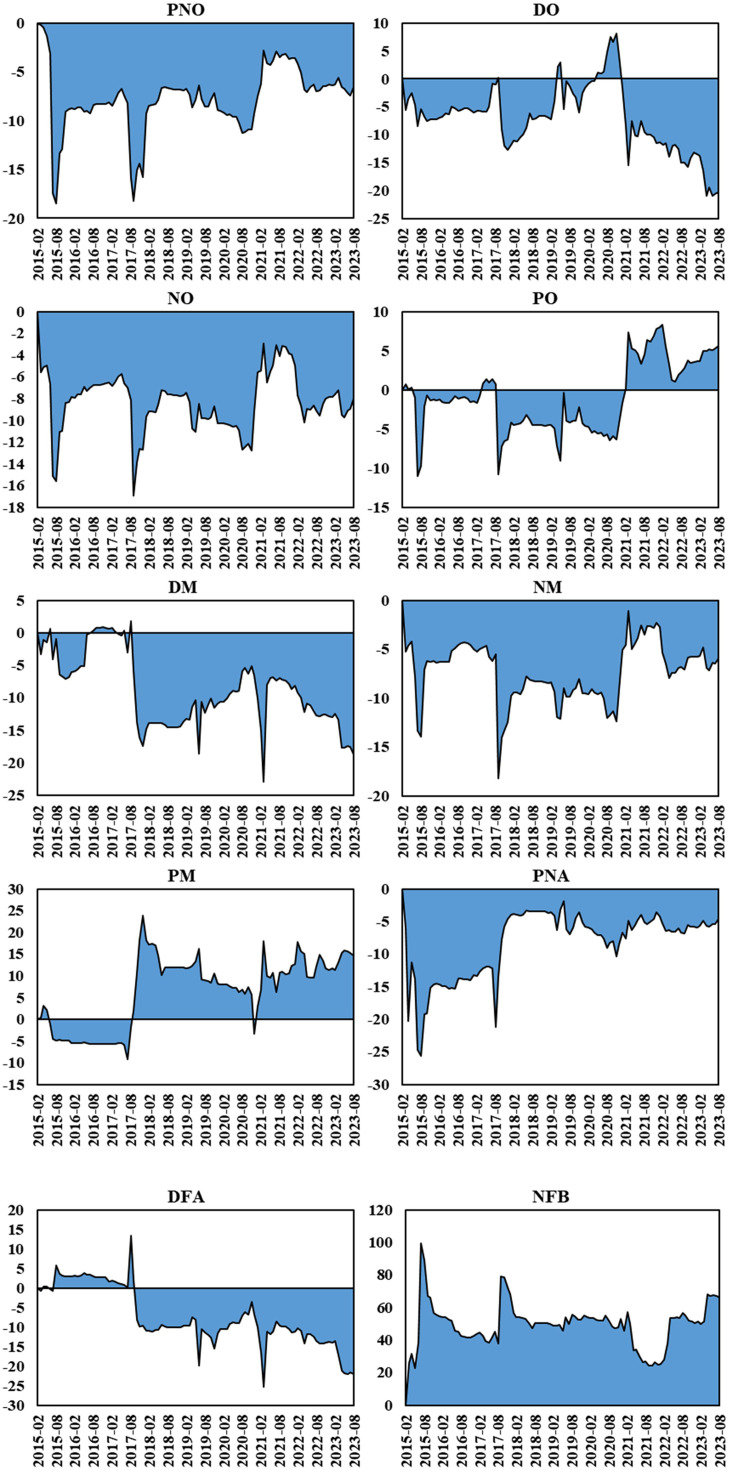
Time-varying net connectivity of various products within China’s rare earth industry chain. Note: This multi-panel figure displays net spillover indices (in percentage terms) for key rare earth products (e.g., PNO, DO, NM, Nd-Fe-B) over February 2015 to August 2023. Each panel’s y-axis shows net connectivity (%), with x-axis as monthly time series labeled by year. Positive values indicate net transmitters, negative as receivers. Shaded regions mark significance, emphasizing shifts like NFB’s consistent transmitter role.

Fig 5 shows the net connectivity of individual rare earth industry chain products over time. Products exhibit either negative net connectivity, acting as receivers of shocks, or positive net connectivity, acting as transmitters of shocks. The findings are consistent with the trends observed in total connectivity and highlight significant time variations.

Key Products as Contributors or Receivers: NFB consistently emerged as the primary contributor to overflow effects, particularly following major clean energy announcements. DFA was a net contributor for most of the sample period but transitioned to a net receiver in late 2017.In early 2021, DO shifted from being a net receiver to a net contributor of spillover effects.

Differences Between Upstream and Midstream Products: Most upstream and midstream products (PNO, DO, NO, DM, NM, and PNA) acted as primary receivers of spillover effects. DM exhibited weaker overflow effects, whereas NFB displayed stronger effects. Compared to other receivers, DM and PNA demonstrated greater hedging capabilities, suggesting potential advantages, particularly concerning NFB.

Changes in the clean energy market significantly enhance the connectivity of the rare earth industry chain, highlighting how market shocks strengthen inter-product relationships. The connectivity patterns among products vary distinctly depending on the stages of rare earth-related policy implementation and the broader impacts of clean energy market dynamics.

As the influence of the clean energy market on rare earth market performance continues to grow, its implications for investors and businesses are expected to further drive the connectivity of the entire rare earth industry chain. Therefore, this study emphasizes the potential impact of China’s clean energy market on the rare earth industry chain, which will be explored further in the subsequent sections.

### ARDL results: Symmetric effects

The changes in the clean energy market represent a key source of risk for the rare earth industry chain’s product markets. To explore the relationship between the Chinese clean energy market and the rare earth industry chain product market, we employed the ARDL and NARDL models. Based on the AIC and BIC, an optimal lag order of 8 was selected.

Before estimation, we conducted unit root tests on the data to ensure their suitability for ARDL and NARDL analysis. The ADF, PP and KPSS tests confirmed that all variables were either integrated at order zero I(0) or one I(1), meeting the data stationarity requirements. Table 5 presents the results of these unit root tests.

**Table 5:** Unit root tests.

Variable	ADF		PP		KPSS	
	Level	1st diff.	Level	1st diff.	Level	1st diff.
TC	−2.274	−9.682***	−2.422	−9.823***	0.624**	0.125
NEI	−7.937***	−10.056***	−7.771***	−36.939***	0.122	0.091
NEVI	−7.916***	−10.132***	−7.832***	−32.148***	0.119	0.104
WIND	−8.655***	−9.390***	−8.573***	−67.633***	0.087	0.072
SOLAR	−7.744***	−9.793***	−7.530***	−42.728***	0.103	0.097
GPR	−5.144***	−8.244***	−5.083***	−31.510***	0.152*	0.078
EPU	−3.113**	−10.437***	−4.572***	−23.030***	0.581**	0.133
TPU	−3.639***	−11.396***	−5.912***	−31.039***	0.093	0.082

Table 6 shows the ARDL regression results and cointegration tests, revealing a long-run relationship between the overall connectivity of the rare earth industry chain and the clean energy market variables (NEI, NEVI, WIND, and SOLAR). The models also incorporate GPR, EPU, and TPU as control variables.

**Table 6:** ARDL regression results.

Panel A: Short-Run Effects			
Model	Variable	Coefficient	Lag
M1	$\Delta NEI_{t-\text{2}}$	0.021***	t-2
	$\Delta NEI_{t-\text{3}}$	0.010*	t-3
	$\Delta NEI_{t-\text{4}}$	0.009**	t-4
	$\Delta NEI_{t-\text{5}}$	0.008**	t-5
	ΔGPR	0.184*	Current
	$\Delta GPR_{t-\text{3}}$	−0.204*	t-3
	ΔEPU	−0.002**	Current
	ΔTPU	0.001***	Current
M2	ΔNEVI	−0.009**	Current
	$\Delta NE{VI}_{t-\text{1}}$	−0.011*	t-1
	$\Delta NEVI_{t-\text{3}}$	−0.007*	t-3
	ΔGPR	0.358***	Current
	$\Delta GPR_{t-\text{2}}$	−0.269*	t-2
	$\Delta GPR_{t-\text{3}}$	−0.340***	t-3
	ΔEPU	−0.001*	Current
	$\Delta EPU_{t-\text{1}}$	0.001*	t-1
	ΔTPU	0.001**	Current
M3	$\Delta WIND_{t-\text{1}}$	0.013*	t-1
	$\Delta WIND_{t-\text{2}}$	0.019***	t-2
	$\Delta WIND_{t-\text{3}}$	0.011**	t-3
	$\Delta WIND_{t-\text{5}}$	0.008**	t-5
	ΔGPR	0.230**	Current
	$\Delta GPR_{t-\text{1}}$	0.289***	t-1
M4	$\Delta SOLAR_{t-\text{1}}$	0.017**	t-1
	$\Delta SOLAR_{t-\text{2}}$	0.023***	t-2
	$\Delta SOLAR_{t-\text{3}}$	0.011**	t-3
	$\Delta SOLAR_{t-\text{4}}$	0.010**	t-4
	$\Delta SOLAR_{t-\text{5}}$	0.008**	t-5
	ΔGPR	0.202*	Current
	$\Delta GPR_{t-\text{3}}$	−0.182*	t-3
	ΔEPU	−0.002**	Current
	ΔTPU	0.001***	Current
Panel B: Long-Run Effects			
Model	Variable	Coefficient	
M1	NEI	−0.195*	
M2	NEVI	0.061	
M3	WIND	−2.724*	
M4	SOLAR	−0.223**	
Panel C: Cointegration Tests			
Model	F-Statistic		
M1	3.568**		
M2	3.102**		
M3	3.386***		
M4	3.927***		

The results indicate that all types of clean energy markets significantly influence the connectivity of the rare earth industry chain:

Short-Run Effects: NEI, NEVI, WIND, and SOLAR exhibit significant positive effects on the overall connectivity. For instance, a 1% increase in NEVI leads to a short-term decrease in connectivity by 0.009%. Meanwhile, NEI, WIND, and SOLAR have significant positive impacts with a lag of one period. Long-Run Effects: Connectivity is negatively impacted by SOLAR, with a 1% increase in solar market activity reducing connectivity by 0.223%. This negative long-run SOLAR effect mirrors Apergis & Apergis (2017)‘s correlations between rare earth prices and renewables [23], but our ARDL framework shows equilibria stabilization, implying rational adjustments not fully captured in their temporal analysis. This suggests that in the long run, participants in the rare earth market respond rationally, aligning their investments with broader market trends.

Cointegration test results for all four models exceed the critical values at the 5% significance level, confirming the presence of a long-run relationship characterized by asymmetry. However, the nonlinearity of these relationships suggests that linear ARDL models may not fully capture the dynamics, warranting further exploration through NARDL models.

The ARDL models highlight the critical role of clean energy market dynamics in shaping the connectivity of rare earth industry chains. The significant short-run impacts emphasize the sensitivity of rare earth markets to immediate changes in clean energy demand. Conversely, the long-run effects suggest that market participants adapt their strategies to align with rational expectations. However, the asymmetric effects and the complexity of these interactions warrant further investigation using nonlinear models to better understand the evolving dynamics.

Economically, the positive short-run effects imply that surges in clean energy indices prompt rapid investor reallocation toward rare earth products, enhancing chain integration and potentially reducing short-term price volatility through diversified demand. In contrast, the negative long-run SOLAR coefficient suggests that as solar markets mature, they stabilize rare earth connectivity by encouraging efficient supply chain adjustments, but this could signal over-reliance on solar demand, advising policymakers to diversify renewable incentives to mitigate risks.

### NARDL results: Asymmetric effects

The NARDL regression results, shown in Table 6, shed light on the intricate relationship between the clean energy market and the connectivity of the rare earth industry chain, supplemented by cointegration test findings. The cointegration tests reject the null hypothesis of no cointegration across all models, indicating a nonlinear long-term linkage between rare earth industry connectivity and key clean energy market variables, including NEI, NEVI, WIND, and SOLAR. These results reveal a complex, asymmetric dynamic between the clean energy sector and the rare earth industry’s overall connectivity.

The NARDL regression models (N1: NEI, N2: NEVI, N3: WIND, and N4: SOLAR) incorporate GPR, EPU, and TPU as control variables. Asymmetric effects are evident in both the short and long run, with the PV market demonstrating significant short- and long-run impacts on rare earth connectivity, while other clean energy markets exhibit primarily long-run asymmetric influences, as confirmed by Wald test results in Table 8.

**Table 7:** NARDL regression results.

Panel A: Short-Run Effects			
Model	Variable	Coefficient	Lag
N1	$\Delta NEI^{-}$	−0.015**	Current
	$\Delta {NEI}_{t-\text{1}}^{+}$	−0.015***	t-1
	ΔGPR	0.322***	Current
	$\Delta GPR_{t-\text{2}}$	−0.285**	t-2
	$\Delta GPR_{t-\text{3}}$	−0.328***	t-3
	$\sum \Omega_{i}^{+}$	−0.015***	Cumulative
	$\sum \Omega_{i}^{-}$	−0.015**	Cumulative
N2	$\Delta NEVI^{-}$	−0.018***	Current
	$\Delta {NEVI}_{t-\text{3}}^{+}$	−0.011*	t-3
	ΔGPR	0.345***	Current
	$\Delta GPR_{t-\text{3}}$	−0.299**	t-3
	ΔTPU	0.001*	Current
	$\sum \Omega_{i}^{+}$	−0.011*	Cumulative
	$\sum \Omega_{i}^{-}$	−0.018***	Cumulative
N3	$\Delta WIND^{-}$	−0.010**	Current
	$\Delta {WIND}_{t-\text{1}}^{+}$	−0.014***	t-1
	ΔGPR	0.340***	Current
	$\Delta GPR_{t-\text{2}}$	−0.300**	t-2
	$\Delta GPR_{t-\text{3}}$	−0.344***	t-3
	$\sum \Omega_{i}^{+}$	−0.014***	Cumulative
	$\sum \Omega_{i}^{-}$	−0.010**	Cumulative
N4	$\Delta SOLAR^{+}$	−0.023***	Current
	$\Delta {SOLAR}_{t-\text{1}}^{+}$	0.034***	t-1
	$\Delta {SOLAR}_{t-\text{2}}^{+}$	0.028***	t-2
	$\Delta {SOLAR}_{t-\text{2}}^{-}$	0.039***	t-2
	$\Delta {SOLAR}_{t-\text{3}}^{+}$	0.018**	t-3
	$\Delta {SOLAR}_{t-\text{4}}^{-}$	0.019**	t-4
	$\Delta {SOLAR}_{t-\text{5}}^{+}$	0.009*	t-5
	ΔGPR	0.207*	Current
	$\Delta GPR_{t-\text{2}}$	−0.408*	t-2
	$\Delta GPR_{t-\text{3}}$	−0.553***	t-3
	$\Delta GPR_{t-\text{4}}$	−0.282*	t-4
	$\Delta GPR_{t-\text{5}}$	−0.305**	t-5
	$\Delta GPR_{t-\text{6}}$	−0.197*	t-6
	$\Delta EPU_{t-\text{3}}$	−0.002**	t-3
	$\Delta EPU_{t-\text{4}}$	−0.002***	t-4
	$\Delta EPU_{t-\text{6}}$	−0.001**	t-6
	ΔTPU	0.001**	Current
	$\Delta TPU_{t-\text{3}}$	0.001**	t-3
	$\Delta TPU_{t-\text{4}}$	0.001**	t-4
	$\sum \Omega_{i}^{+}$	0.066*	Cumulative
	$\sum \Omega_{i}^{-}$	0.058**	Cumulative
Panel B: Long-Run Effects			
Model	Variable	Coefficient	
N1	NEI+	0.002	
	NEI-	0.016	
N2	NEVI+	0.016	
	NEVI-	0.029	
N3	WIND+	−0.006	
	WIND-	0.006	
N4	SOLAR+	−0.205***	
	SOLAR-	−0.192***	
Panel C: Cointegration Tests			
Model	F-Statistic		
N1	2.878**		
N2	2.655**		
N3	3.225**		
N4	4.287***		

**Table 8:** Asymmetric response tests.

Variables	Long-term asymmetric test		Short-term asymmetric test	
	F-statistic	Chi-square	F-statistic	Chi-square
NEI	8.613***	8.613***	0.035	0.035
NEVI	6.431**	6.431**	0.192	0.192
WIND	10.001***	10.001***	0.139	0.139
SOLAR	7.351***	7.351***	6.869**	6.869***

In the short run, the results for Model N1 indicate that a 1% increase (decrease) in NEI reduces (raises) rare earth industry chain connectivity by 0.015%, reflecting symmetric short-run effects. Similar patterns are observed for NEVI and WIND. However, the impact of SOLAR stands out with pronounced asymmetry: a 1% increase (decrease) in SOLAR raises (lowers) connectivity by 0.066% (0.058%). These SOLAR asymmetries extend Ding et al. (2024)‘s positive short-term effects during COVID [22], but our NARDL reveals stronger disruptions from negatives. These findings underscore the significant role of clean energy markets in shaping short-term dynamics within the rare earth industry chain. For instance, rising returns in the new energy vehicle and wind energy markets encourage investors to stabilize rare earth material industrial chains to secure consistent returns, thereby reducing market volatility, as per contagion theory in interconnected markets [37]. Conversely, declining returns can heighten investor risk aversion, leading to temporary disruptions. Nevertheless, the optimistic market outlook and strong government backing for clean energy help cushion the adverse effects on rare earth market volatility. In the PV market, government-led initiatives to integrate photovoltaics into residential infrastructure have closely tied its growth to daily life. When PV returns increase, investors intensify short-term investments in related rare earth products, boosting connectivity. On the other hand, declines in PV returns trigger minimal short-term adjustments due to long-term confidence in the market’s resilience and potential.

Table 7 also presents the long-run coefficients of clean energy markets on rare earth industry connectivity. Models N1, N2, and N3 suggest that NEI, NEVI, and WIND exert no significant long-term effects, highlighting rational investor behavior and the stabilizing influence of long-term expectations on market volatility. In contrast, Model N4 (SOLAR) reveals pronounced long-term asymmetry: a 1% increase (decrease) in SOLAR reduces (raises) connectivity by 0.205% (0.192%). This long-run finding builds on Gao et al. (2024)‘s higher-order connectedness, but differentiates by showing positive shocks’ saturation effects [27], unique to China’s PV dominance. This finding underscores the pivotal role of the PV market in stabilizing the rare earth industrial chain. Positive shocks, such as rising PV returns, mitigate risk spillovers by bolstering industrial chain stability through integrated supply chains under electrification [17]. Conversely, persistent negative shocks, like sustained declines in returns, prompt gradual portfolio adjustments, leading to increased volatility.

Among clean energy markets, the PV market has the most substantial influence on rare earth connectivity. Negative short-term shocks in PV returns have a more pronounced impact than positive ones, although their long-term effects are muted. In contrast, positive long-term shocks yield greater connectivity benefits, highlighting the PV market’s stabilizing influence. Table 8 confirms the statistical significance of these asymmetric relationships, with PV market returns closely tied to rare earth industrial chains, further emphasizing the sector’s central role in the global energy transition.

Overall, variations in China’s clean energy market demonstrate a mix of nonlinear and asymmetric effects on rare earth industry connectivity. Rapid fluctuations in NEV and wind energy markets can influence investor sentiment, creating temporary spillovers. However, robust government support fosters rational investment decision-making, reducing overall volatility. The PV market stands out due to its strong integration into daily life and policy prioritization, amplifying its impact on rare earth connectivity. Over time, the clean energy market has promoted greater transparency and rational investor behavior, gradually diminishing the frequency and intensity of connectivity disruptions within the rare earth industry chain.

From an economic viewpoint, the asymmetric SOLAR short-run effects indicate that positive shocks foster bullish market behavior, driving investment inflows and tighter chain linkages, while negative shocks trigger risk-averse pullbacks, exacerbating volatility—consistent with contagion effects [37]. In the long run, the stronger negative impact from positive SOLAR changes implies market saturation, where excess demand leads to overcapacity and reduced connectivity, urging policies like strategic reserves to balance supply [10].

### Robustness discussion

To validate the findings, we compare ARDL and NARDL results. ARDL reveals symmetric short-run positives for clean energy indices and a consistent negative long-run SOLAR effect, aligning with NARDL’s asymmetric SOLAR impacts, suggesting robustness in solar’s stabilizing role. Consistencies include non-significant long-run effects for NEI/NEVI/WIND across models, indicating rational market adaptation. Differences highlight NARDL’s capture of asymmetries, e.g., stronger short-run SOLAR positives, underscoring nonlinearity. Sub-sample tests (pre/post-2020) and alternative proxies (e.g., ICRG for GPR) confirm these patterns, with minor volatility variations, affirming the models’ reliability in depicting clean energy-rare earth dynamics.

## Conclusions

In summary, this study reveals high interconnectivity in China’s rare earth markets, with dynamic spillovers driven by clean energy fluctuations, particularly PV’s asymmetric effects that stabilize chains in the long run while causing short-run parallels. NEV and WE show inverse short-run variations, underscoring sector-specific dynamics.

This study examines how China’s clean energy market affects the interconnectivity of rare earth industry chain product markets. By incorporating multiple key products of the Chinese rare earth sector and employing a TVP-VAR model, we identified dynamic spillover effects among these products. Subsequently, the ARDL and NARDL models were used to investigate how four indicators of the Chinese clean energy market asymmetrically influence connectivity within the rare earth industry chain. The empirical results indicate a high degree of interconnectedness among the product markets of the Chinese rare earth industry chain, with the PV market exerting a particularly strong impact. NFB was identified as a primary spillover contributor, while praseodymium-neodymium alloys PNA and dysprosium metal DM serve as effective hedges against market fluctuations from other rare earth products. These patterns directly support China’s “Made in China 2025” initiative, which emphasizes technological self-reliance in high-tech sectors like renewables, by demonstrating how clean energy demand strengthens rare earth chain resilience. Similarly, the asymmetries in PV effects align with the 2060 carbon neutrality goals, as they reveal mechanisms for stabilizing supply chains essential to scaling solar technologies and reducing emissions.

In the short run, changes in the NEV and WE markets tend to produce inverse variations in overall connectivity, whereas fluctuations in the PV market exert parallel impacts on rare earth product markets. This occurs because NEV and WE demand shocks often trigger rapid but opposing investor responses—e.g., positive shocks encourage hedging that temporarily fragments chains, while negative ones consolidate them for risk mitigation—reflecting supply rigidities in rare earth extraction. Over the long term, positive PV market developments reduce the spread risk among rare earth products, highlighting the sector’s importance for stabilizing investor sentiment. This stabilization arises from PV’s integration into national grids, fostering predictable demand that smooths volatility, as opposed to the cyclical nature of NEV/WE. In particular, investors are more sensitive to positive information regarding the PV market—such as technological innovation and supportive policies—than to negative news, due to PV’s alignment with fixed infrastructure investments under “Made in China 2025”. Moreover, while NE, NEV, and WE indicators also show asymmetric impacts on connectivity, these effects are generally less pronounced compared to those from the PV market, implying PV’s outsized role in driving the 2060 neutrality agenda through reliable rare earth linkages.

Building on these results, we evaluate the hypotheses in detail, linking back to the theoretical framework of bullwhip, capacity constraints, cost compensation, and contagion effects [28,30,31]. H1 is partially accepted: Short-run positive enhancements from clean energy align with bullwhip amplification (Pathway 1), as surges boost spillovers, but long-run weakenings reflect capacity adjustments (Pathway 2), consistent with market stabilization. H2 is accepted: Asymmetries, with negatives stronger, tie to contagion (Pathway 4, [37]), where declines exacerbate disruptions via risk aversion more than positives integrate via demand-pull. H3 is accepted: PV’s role supports optimization, linked to cost compensation (Pathway 3, [31]), enabling recommendations for subsidies and reserves to mitigate risks through subsidies and reserves.

From a practical standpoint, the findings underscore the importance of tracking developments in China’s clean energy sector, especially the PV market, to better understand and manage risks associated with rare earth investments. In the short term, it may be beneficial for investors to hedge against panic by focusing on products that can buffer against adverse spillovers, such as dysprosium metal or praseodymium-neodymium alloys, when risks escalate in other product markets. Over the long term, as risk transmission gradually decreases, monitoring the growth potential of the PV market allows investors to adjust their rare earth portfolios, diversify risks, and enhance returns. Regulatory agencies and policymakers can leverage these insights to promote greater stability. Tailored support for the PV industry—through incentives such as fiscal subsidies, tax breaks, and R&D funding—can nurture further innovation and build investor confidence in upstream rare earth product markets. Transparent information disclosure, combined with effective management of strategic reserves and supply-chain monitoring, could mitigate volatility and prevent supply bottlenecks as clean energy demand continues to expand. To enhance market transparency in clean energy sectors, authorities could mandate real-time reporting of PV production data and subsidies, reducing information asymmetries that exacerbate short-run volatility. Balancing export control of rare earth materials with renewable development goals requires integrated planning, such as aligning the 2020 Export Control Law with 2060 neutrality targets to ensure domestic supply for PV without stifling global trade. Promoting R&D investment to reduce dependency on rare earth imports could involve targeted grants under “Made in China 2025,” focusing on recycling technologies and substitutes to diversify sources and stabilize long-run connectivity.

Although this study reveals significant relationships between the clean energy and rare earth markets, it leaves room for further exploration. Future research could probe how variations in clean energy markets affect different phases of shocks in rare earth product markets and delve deeper into the channels through which investor sentiment responds to media reports on emerging technologies or regulatory changes. Additionally, our analysis focuses on the time-varying impacts of market returns; subsequent work may consider volatility and frequency-domain effects to enrich the understanding of these inter-market linkages. By integrating external factors such as global trade disputes, technological breakthroughs in substitutes or recycling methods, and macroeconomic shocks, further studies can help refine these findings and offer more comprehensive insights for industry stakeholders and policy designers. Overall, the results highlight the increasing significance of the PV market for stabilizing risk transmission within the rare earth industry chain and provide a basis for informed decision-making in both investment and policy contexts.

This study has several notable strengths. First, the integration of dynamic econometric models—such as TVP-VAR for time-varying spillovers, ARDL for long-run equilibria, and NARDL for asymmetries—provides a comprehensive framework to analyze evolving market interactions, surpassing traditional static approaches. Second, the novel dataset, combining monthly returns from 10 rare earth products with four clean energy indices and controls like GPR and EPU over 2015–2023, captures real-time dynamics during pivotal policy eras. Third, the findings offer high policy relevance, directly informing China’s “Made in China 2025” and 2060 carbon neutrality goals by highlighting PV’s stabilizing role in rare earth chains. However, several limitations should be acknowledged. The sample size of 103 monthly observations, while covering key events, may limit statistical power in detecting subtle asymmetries, particularly in sub-period analyses. Data frequency mismatch arises as monthly aggregates could overlook high-frequency intraday or weekly volatility in rare earth prices driven by real-time news. Potential omitted variables, such as advancements in rare earth substitutes or macroeconomic indicators like global inflation, might bias estimates by not fully capturing external influences. Additionally, the focus on China’s domestic markets excludes international spillovers, such as those from US rare earth policies or EU green deals, potentially underestimating global interdependencies.

Future studies could extend the models to explore cross-country spillovers, such as US-China rare earth dependencies amid trade tensions, using global TVP-VAR frameworks. Nonlinear causality tests (e.g., Granger extensions) might uncover directional influences under regime shifts, while volatility modeling (e.g., GARCH integrations) could assess risk persistence in frequency domains, providing deeper insights into global clean energy transitions.

## Supporting information

S1 Text **S1 File. Supporting information 1.** Dataset of rare earth prices from Wind Database (Shanghai Nonferrous Metals Network), including Praseodymium-Neodymium Oxide, Dysprosium Oxide, Neodymium Oxide, Praseodymium Oxide, Dysprosium Metal, Neodymium Metal, Praseodymium Metal, Pr-Nd Alloy, Dy-Fe Alloy, and Nd-Fe-B. **S2 File. Supporting information 2.** Dataset of clean energy market indices and economic uncertainty indices from CSI Index Website (www.csindex.com.cn) and Economic Policy Uncertainty Website (www.policyuncertainty.com), including New Energy Index, New Energy Vehicle Index, Wind Power Industry Index, Photovoltaic Industry Index, Geopolitical Risk Index, Economic Policy Uncertainty Index, and Trade Policy Uncertainty Index.
(ZIP)

## References

[1] World Energy Outlook 2023. World Energy Outlook. OECD. 2023. doi: 10.1787/827374a6-en

[2] Bernstein L. Climate Change 2007: Synthesis Report: A Report of the Intergovernmental Panel on Climate Change. Geneva: IPCC. 2008.

[3] Sovacool BK, Sidortsov RV, Jones BR. Energy Security, Equality and Justice. Routledge. 2013. https://www.taylorfrancis.com/books/9781135074197

[4] Binnemans K, Jones PT, Blanpain B, Van Gerven T, Yang Y, Walton A. Recycling of rare earths: a critical review. J Clean Prod. 2013;51:1–22.

[5] Golev A, Scott M, Erskine PD, Ali SH, Ballantyne GR. Rare earths supply chains: Current status, constraints and opportunities. Resources Policy. 2014;41:52–9. doi: 10.1016/j.resourpol.2014.03.004

[6] Massari S, Ruberti M. Rare earth elements as critical raw materials: Focus on international markets and future strategies. Resources Policy. 2013;38(1):36–43. doi: 10.1016/j.resourpol.2012.07.001

[7] Olivetti EA, Ceder G, Gaustad GG, Fu X. Lithium-Ion Battery Supply Chain Considerations: Analysis of Potential Bottlenecks in Critical Metals. Joule. 2017;1(2):229–43. doi: 10.1016/j.joule.2017.08.019

[8] Wübbeke J, Meissner M, Zenglein MJ, Ives J, Conrad B. Made in China 2025: the making of a high-tech superpower and consequences for industrial countries. Berlin: Mercator Institute for China Studies. 2016. https://merics.org/sites/default/files/2020-04/Made%20in%20China%202025.pdf

[9] Sprecher B, Xiao Y, Walton A, Speight J, Harris R, Kleijn R, et al. Life cycle inventory of the production of rare earths and the subsequent production of NdFeB rare earth permanent magnets. Environ Sci Technol. 2014;48(7):3951–8. doi: 10.1021/es404596q 24576005

[10] Jowitt SM, Werner TT, Weng Z, Mudd GM. Recycling of the rare earth elements. Current Opinion in Green and Sustainable Chemistry. 2018;13:1–7. doi: 10.1016/j.cogsc.2018.02.008

[11] Schüler D, Buchert M, Liu R, Dittrich S, Merz C. Study on rare earths and their recycling. Darmstadt: Öko-Institut e.V. 2011. https://www.oeko.de/oekodoc/1112/2011-003-en.pdf

[12] Shuai J, Peng X, Zhao Y, Wang Y, Xu W, Cheng J, et al. A dynamic evaluation on the international competitiveness of China’s rare earth products: An industrial chain and tech-innovation perspective. Resources Policy. 2022;75:102444. doi: 10.1016/j.resourpol.2021.102444

[13] Zuo Z, McLellan BC, Li Y, Guo H, Cheng J. Evolution and insights into the network and pattern of the rare earths trade from an industry chain perspective. Resources Policy. 2022;78:102912. doi: 10.1016/j.resourpol.2022.102912

[14] Leng Z, Sun H, Cheng J, Wang H, Yao Z. China’s rare earth industry technological innovation structure and driving factors: A social network analysis based on patents. Resources Policy. 2021;73:102233. doi: 10.1016/j.resourpol.2021.102233

[15] Zhang H, Wang X, Tang J, Guo Y. The impact of international rare earth trade competition on global value chain upgrading from the industrial chain perspective. Ecol Econ. 2022;198:107472.

[16] Xia Q, Du D, Cao W, Li X. Who is the core? Reveal the heterogeneity of global rare earth trade structure from the perspective of industrial chain. Resources Policy. 2023;82:103532. doi: 10.1016/j.resourpol.2023.103532

[17] Severson MH, Nguyen RT, Ormerod J, Williams S. An integrated supply chain analysis for cobalt and rare earth elements under global electrification and constrained resources. Resour Conserv Recycl. 2023;189:106761. doi: 10.1016/j.resconrec.2023.106761

[18] Xu C, Xu Y, Gao C. Reevaluating multilayered trade patterns and risk contagion in the global rare earth market. Miner Econ. 2025;:0–19.

[19] International Energy Agency. Renewables 2023: analysis and forecast to 2028. Paris: IEA. 2024. https://www.iea.org/reports/renewables-2023

[20] World energy investment 2023. Paris: IEA. 2023. https://www.iea.org/reports/world-energy-investment-2023

[21] Alonso E, Sherman AM, Wallington TJ, Everson MP, Field FR, Roth R, et al. Evaluating rare earth element availability: a case with revolutionary demand from clean technologies. Environ Sci Technol. 2012;46(6):3406–14. doi: 10.1021/es203518d 22304002

[22] Ding Q, Huang J, Chen J, Luo X. Climate warming, renewable energy consumption and rare earth market: Evidence from the United States. Energy. 2024;290:130276. doi: 10.1016/j.energy.2024.130276

[23] Apergis E, Apergis N. The role of rare earth prices in renewable energy consumption: The actual driver for a renewable energy world. Energy Economics. 2017;62:33–42. doi: 10.1016/j.eneco.2016.12.015

[24] Fritz M, Schiefer G. System dynamics and innovation in food networks. Br Food J. 2009;111(8). doi: 10.1108/bfj.2009.070111haa.001

[25] Madaleno M, Taskin D, Dogan E, Tzeremes P. A dynamic connectedness analysis between rare earth prices and renewable energy. Resources Policy. 2023;85:103888. doi: 10.1016/j.resourpol.2023.103888

[26] Ye R, Gong J, Xia X. Trading risk spillover mechanism of rare earth in China: new perspective based on time-varying connectedness approach. Systems. 2023;11(4):168–90.

[27] Gao W, Wei J, Zhang H, Zhang H. The higher-order moments connectedness between rare earth and clean energy markets and the role of geopolitical risk:New insights from a TVP-VAR framework. Energy. 2024;305:132280. doi: 10.1016/j.energy.2024.132280

[28] Lee HL, Padmanabhan V, Whang S. Information distortion in a supply chain: the bullwhip effect. Manag Sci. 1997;43(4):546–58.

[29] McFarland RG, Bloodgood JM, Payan JM. Supply Chain Contagion. J Mark. 2008;72(2):63–79.

[30] Ponte B, Wang X, De La Fuente D, Disney SM. Exploring nonlinear supply chains: the dynamics of capacity constraints. Int J Prod Res. 2017;55(14):4053–67.

[31] Shan F, Dai X, Men J, Yang F. Coordinating a decentralized supply chain with capacity cost compensation. RAIRO-Oper Res. 2021;55:S1789–802. doi: 10.1051/ro/2020056

[32] Bray RL, Mendelson H. Information transmission and the bullwhip effect: An empirical investigation. Manag Sci. 2012.

[33] Metters R. Quantifying the bullwhip effect in supply chains. J Oper Manag. 1997;15(2):89–100.

[34] Yang Y, Lin J, Liu G, Zhou L. The behavioural causes of bullwhip effect in supply chains: A systematic literature review. Int J Prod Econ. 2021;236:108120.

[35] Zhu T, Balakrishnan J, Da Silveira GJC. Bullwhip effect in the oil and gas supply chain: A multiple-case study. Int J Prod Econ. 2020;224:107548.

[36] Wang J, Zhou Z, Yu M. Pricing models in a sustainable supply chain with capacity constraint. J Clean Prod. 2019;222:57–76.

[37] Filbeck G, Kumar S, Liu J, Zhao X. Supply chain finance and financial contagion from disruptions - evidence from the automobile industry. Int J Phys Distrib Logist Manag. 2016;46(4).

[38] Vincent Eagan J, Subrahmanyam V, Alli K. Research note: neural network analysis of dividend policy. Managerial Finance. 1999;25(6):44–56. doi: 10.1108/03074359910766000

[39] Wang X-Q, Wu T, Zhong H, Su C-W. Bubble behaviors in nickel price: What roles do geopolitical risk and speculation play?. Resources Policy. 2023;83:103707. doi: 10.1016/j.resourpol.2023.103707

[40] Jyothi RK. Rare-Earth Metal Recovery for Green Technologies. Springer International Publishing. 2020. doi: 10.1007/978-3-030-38106-6

[41] Jyothi RK, Thenepalli T, Ahn JW, Parhi PK, Chung KW, Lee JY. Review of rare earth elements recovery from secondary resources for clean energy technologies: Grand opportunities to create wealth from waste. J Clean Prod. 2020;267:122048.

[42] Hanif W, Mensi W, Gubareva M, Teplova T. Impacts of COVID-19 on dynamic return and volatility spillovers between rare earth metals and renewable energy stock markets. Resources Policy. 2023;80:103196. doi: 10.1016/j.resourpol.2022.103196

[43] Primiceri GE. Time Varying Structural Vector Autoregressions and Monetary Policy. The Review of Economic Studies. 2005;72(3):821–52. doi: 10.1111/j.1467-937x.2005.00353.x

[44] Antonakakis N, Gabauer D, Gupta R. International monetary policy spillovers: evidence from a time-varying parameter vector autoregression. Int Rev Financ Anal. 2019;65:101382.

[45] Nakajima J, Kasuya M, Watanabe T. Bayesian analysis of time-varying parameter vector autoregressive model for the Japanese economy and monetary policy. J Jpn Int Econ. 2011;25(3):225–45.

[46] Wen F, Min F, Zhang Y, Yang C. Crude oil price shocks, monetary policy, and China’s economy. Int J Fin Econ. 2018;24(2):812–27. doi: 10.1002/ijfe.1692

[47] Antonakakis N, Chatziantoniou I, Gabauer D. Refined Measures of Dynamic Connectedness based on Time-Varying Parameter Vector Autoregressions. JRFM. 2020;13(4):84. doi: 10.3390/jrfm13040084

[48] Diebold FX, Yılmaz K. On the network topology of variance decompositions: Measuring the connectedness of financial firms. J Econom. 2014;182(1):119–34.

[49] Pesaran MH, Shin Y, Smith RJ. Bounds testing approaches to the analysis of level relationships. J Appl Econom. 2001;16(3):289–326.

[50] Shin Y, Yu B, Greenwood-Nimmo M. Modelling Asymmetric Cointegration and Dynamic Multipliers in a Nonlinear ARDL Framework. Festschrift in Honor of Peter Schmidt. Springer New York. 2014. 281–314. doi: 10.1007/978-1-4899-8008-3_9

[51] Van Treeck T. Reconsidering the investment–profit nexus in finance‐led economies: an ardl‐based approach. Metroeconomica. 2008;59(3):371–404. doi: 10.1111/j.1467-999x.2008.00312.x

[52] Davis SJ, Liu D, Sheng XS. Economic policy uncertainty in China since 1949: the view from mainland newspapers. Chicago: University of Chicago Booth School of Business. 2019. https://static1.squarespace.com/static/5e2ea3a8097ed30c779bd707/t/5f7f49d054a84229354fe9ab/1602177496854/EPU.in.China%2C.View.from.Mainland.Newspapers%2C.August.2019.pdf

[53] Broock WA, Scheinkman JA, Dechert WD, LeBaron B. A test for independence based on the correlation dimension. Econom Rev. 1996;15(3):197–235.

[54] Bouri E. Spillovers in the joint system of conditional higher-order moments: US evidence from green energy, brown energy, and technology stocks. Renewable Energy. 2023;210:507–23. doi: 10.1016/j.renene.2023.04.006

